# Cost-effectiveness of atosiban versus placebo for threatened preterm birth (APOSTEL 8 trial)

**DOI:** 10.1371/journal.pone.0347475

**Published:** 2026-11-05

**Authors:** Larissa van der Windt, Martijn Oudijk, Job Klumper, Tobias Nijman, Judith Bosmans, Ben Willem Mol, Joris van der Post, Carolien Roos, Stavros Petrou, Kate Walker

**Affiliations:** 1 Department of Obstetrics and Gynaecology, Amsterdam UMC location University of Amsterdam, Amsterdam, Netherlands; 2 Amsterdam Reproduction and Development Research Institute, Amsterdam, Netherlands; 3 Department of Obstetrics and Gynaecology, Amsterdam UMC, Location Vrije Universiteit Amsterdam, Amsterdam, Netherlands; 4 Department of Obstetrics and Gynaecology, University Medical Center Utrecht, Utrecht University, Utrecht, Netherlands; 5 Department of Health Sciences, Faculty of Science, Vrije Universiteit Amsterdam, Amsterdam Public Health Research Institute, Amsterdam, Netherlands; 6 Department of Obstetrics and Gynaecology, Monash University, Clayton, Australia; 7 Nuffield Department of Primary Care Health Sciences, University of Oxford, Oxford, United Kingdom; 8 Centre for Perinatal Research University of Nottingham, Queen’s Medical Centre, Nottingham, England; Korea University Medicine, KOREA, REPUBLIC OF

## Abstract

**Background:**

To assess the cost-effectiveness of treatment with atosiban compared to placebo for threatened preterm birth from 30^+0^ to 33^+6^ weeks of gestation.

**Methods:**

We performed an economic evaluation from a societal perspective alongside the multicentre randomised controlled APOSTEL 8 trial, including 755 participants with threatened preterm birth from 30^+0^ to 33^+6^ weeks of gestation who were randomised to atosiban or placebo, of whom 752 (884 infants) were included in the intention-to-treat analysis. Costs, collected between randomisation and three months corrected child age, were measured using a combination of hospital records and the self-reported iMTA Medical and Productivity Cost Questionnaires and estimated at infant level. The main outcome measure was cost-effectiveness expressed in terms of incremental cost per severe adverse neonatal outcome prevented. Multiple imputation by chained equations was used to handle missing data. Non-parametric bootstrapping was used to estimate statistical uncertainty surrounding incremental cost-effectiveness outcomes.

**Findings:**

The difference in mean costs between the atosiban group (€40,743.1) and the placebo group (€40,793.6) was not statistically significant (mean difference: -€50.5; 95% confidence interval (CI) −4037.6 to 3936.7). The incremental cost per severe adverse neonatal outcome prevented was dominant (-€5286.6), falling in the south-east quadrant of the cost-effectiveness plane, indicating cost-savings and reduced severe adverse neonatal outcomes by atosiban. However, the bootstrapped cost-effect pairs were distributed across all four quadrants of the cost-effectiveness plane, indicating high uncertainty surrounding cost-effectiveness estimates. The probability of cost-effectiveness of atosiban reached 50% at a willingness to pay threshold of €33,023.3 per severe adverse neonatal outcome prevented.

**Interpretation:**

This economic evaluation did not demonstrate that atosiban is a cost-effective treatment for threatened PTB from 30^+0^ to 33^+6^ weeks gestation. In addition to the absence of clinical benefit, our findings show no economic advantage, further discouraging its routine use at this gestational age. These results contribute to optimizing evidence-based management of threatened preterm birth.

## Introduction

An estimated 13.4 million babies are born preterm worldwide, representing 10% of all pregnancies [1]. Preterm birth (PTB) can result in severe short- and long-term complications, placing a substantial burden on global health and subsequently public sector resources [1,2]. In the United Kingdom, PTB was estimated to cost the public sector £2.95 billion (€3.69 billion) in 2006, with a surviving preterm child to 18 years costing £22,850 (€28,579) more than a term-born peer by age [3]. These incremental costs were almost three fold higher in very preterm children (<33 weeks of gestation) and four times higher in extremely preterm children (<28 weeks of gestation) [3]. This illustrates the importance to consider both clinical and economical outcomes in management of PTB. Ultimately, this will allow policymakers, clinicians and other stakeholders to identify the most cost-effective healthcare strategies to maximize health benefits at a population level [4].

In case of threatened PTB, tocolytics can delay birth to allow for corticosteroid administration which significantly improve neonatal outcomes [5–8]. However, a benefit of tocolytics on neonatal outcomes remains unproven [9]. Therefore, the international randomised controlled APOSTEL 8 (Assessment of Perinatal Outcome by use of Specific Tocolysis in Early Labour) trial was conducted, assessing superiority of tocolytic treatment with atosiban over placebo for threatened PTB from 30^+0^ to 33^+6^ weeks of gestation on neonatal outcomes. Neonatal outcomes did not significantly differ between treatment groups [10]. The trial concluded that standardised use of tocolytics as treatment for threatened PTB from 30^+0^ to 33^+6^ weeks’ gestation is insufficiently supported by current evidence. However, interventions can be cost-effective even in absence of clinical effectiveness [11]. In the APOSTEL 8 trial, prolongation of pregnancy beyond 48 hours was significantly more frequent in the atosiban group compared to the placebo group. This could result in differences in societal costs or healthcare resource utilisation, which were not assessed in the main trial. Economic evaluations can detect subtle differences that may not be immediately apparent through clinical endpoints, but still have important economic implications. Given that PTB is a major contributor of lost human capital across all ages, it is important to assess cost-effectiveness of PTB interventions. Yet, such data remains underreported in literature [12]. Therefore, we conducted an economic evaluation alongside the APOSTEL 8 trial, assessing cost-effectiveness of atosiban for threatened PTB from 30^+0^ to 33^+6^ weeks of gestation until three months corrected child age from a societal perspective.

## Materials and methods

### Study design and participants

We conducted an economic evaluation alongside the randomised double-blind placebo-controlled APOSTEL 8 trial, which was conducted in 24 Dutch hospitals (17 secondary care and seven academic hospitals) collaborating in the Dutch Consortium for Healthcare Evaluation and Research in Obstetrics and Gynecology (NVOG consortium), and one hospital each in England and Ireland. The results of the APOSTEL 8 trial have been published elsewhere [10]. The economic evaluation was pre-specified as part of the primary objective of the study and its methods were described in the original trial protocol (paragraph entitled cost-effectiveness analysis) [13]. The Medical Research Ethics Committee (MREC) of the Amsterdam University Medical Centre approved the study and the boards of all participating clinical centres approved local execution (METC_2017_101). The trial was registered on the EudraCT registry on July 6^th^ 2017 (2017-001007-72) and was funded by ZonMw (#848041004), the Dutch Health Research and Development Organisation. The funder had no influence on the design, conduct of the trial and was not involved in data collection, analysis, interpretation or the writing of the manuscript

Between December 4, 2017, and July 24, 2023, 755 participants with a singleton or twin pregnancy with threatened PTB from 30^+0^ to 33^+6^ weeks of gestation were randomised to atosiban (N = 377) or placebo (N = 378) after written informed consent was obtained. Threatened PTB was defined as uterine contractions with one of the following: cervical length < 15 mm; cervical length 15–30 mm and a positive fibronectin or Actim Partus test; solely a positive fibronectin test or Actim Partus test if transvaginal ultrasound cervical length measurement was not part of local protocol; or ruptured amniotic membranes. Study medication was administered during hospital admission for 48 hours and both participants and investigators were blinded to trial allocation. The primary outcome of the trial was a composite adverse neonatal outcome, defined as perinatal mortality or the occurrence of at least one of the following: bronchopulmonary dysplasia (BPD) [14]; periventricular leukomalacia (PVL)> grade 1 [15]; intraventricular haemorrhage > grade 2; [16] necrotising enterocolitis (NEC)> Bell’s stage 1 [17]; retinopathy of prematurity (ROP)> grade 2 or needing laser therapy; and culture proven sepsis. Full details of the clinical trial including its sample size requirements, randomisation and intervention procedures and clinical outcomes are reported in the study protocol [10,13].

### Economic evaluation

The primary cost-effectiveness outcome in this economic evaluation was incremental cost per severe adverse neonatal outcome prevented. This is in line with the primary outcome of the main APOSTEL 8 trial, since the primary goal of tocolytics should be the improvement of neonatal outcomes [10]. As the clinical effectiveness measure was a neonatal outcome, costs were calculated per neonate (N = 884, more details are described under Statistical analysis and S1 Table in S1 File supporting information). We adopted a societal perspective, meaning that both medical and non-medical costs were included in the economic evaluation. Medical costs included intervention, admission and other healthcare utilisation costs. Non-medical costs included both direct costs from trial participants (e.g., travel expenses), lost productivity and time spent by caregivers providing informal care for the participant. Costs were collected from randomisation until three months of corrected child age. Dutch unit costs were used for all participants, including those from England and Ireland, since only 14 out of 755 (1.9%) participants were recruited from England or Ireland. Also, there are many similarities in these health care systems, including universal health coverage, a comparable level of prosperity and funding of healthcare by a mix of private and public payments [18]. Unit costs were expressed in Euros and valued in 2023 prices [19]. Discounting of costs and clinical outcomes was unnecessary since the follow-up period for each participant was limited to a maximum of six months [20].

### Measurement of resource use

Resource use was collected during three phases: antepartum/intrapartum period, postpartum admission period and post-hospital discharge period until three months of corrected child age (S2 Table in S1 File supporting information). Resource use during the antepartum/intrapartum period and postpartum admission period was collected in the trial Case Report Forms that were also used for the clinical trial. During the antepartum/intrapartum periods, this included: medication use, epidural placement during labour, mode of birth, manual placental removal, at home telemonitoring and maternal admissions. Mode of birth was specified as spontaneous, breech, twin or instrumental vaginal birth, or caesarean section. For twin pregnancies, when mode of birth was similar for both babies (e.g., both spontaneous vaginal), this was counted as one vaginal twin birth. When mode of birth differed between twins, both birth modes were counted, which is in line with the methods of the cost-effectiveness of the APOSTEL 3 study.{Nijman, 2019 #9587} Resource use during the postpartum admission period included: maternal and neonatal admissions, neonatal transfers to another hospital and neonatal operating costs. Hospital admission costs were differentiated based on department of admission; general ward, medium care unit (intermediate care or neonatal level 1 care) or intensive care unit (neonatal level 2–4 care). Resource use during the postpartum period after hospital discharge was collected by the adapted versions of the iMTA Medical and Productivity Cost Questionnaires (iMCQ and iPCQ), sent to trial participants at three months of corrected child age [21]. Resource use concerned healthcare use, informal care defined as the number of extra hours spent by surrounding relations and productivity losses. Productivity losses included paid and unpaid work losses, and education losses (e.g., hours not able to attend classes), were captured from trial entry until three months corrected child age. Lost productivity was valued using the friction cost method, which assumes absent workers are replaced after an adaptation period [19,22]. The friction period was set at 19.40 weeks based on Dutch costing guidelines, limiting productivity loss to that time. Absenteeism from paid work was adjusted for official maternity leave [19]. Travel costs were estimated based on average travel distances retrieved from the Dutch guidelines on costs in healthcare services [19].

### Valuation of resource use

Unit costs were estimated using different methods and sources in line with recent Dutch guidelines on costing of healthcare services (S1 Table in S1 File supporting information) [19,23]. The costs of the placebo were set at €0. As participants were recruited from both secondary and academic hospitals, unit costs were retrieved from the financial departments of one hospital of each type and averaged. This approach was not used for NICU costs, as NICU care is only situated in academic hospitals in the Netherlands. Admission costs were determined using independent top-down gross costing, excluding non-applicable items. Some healthcare costs (e.g., general practitioner or midwives visits) were retrieved from the Dutch guidelines on costs for healthcare services and inflated to 2023 prices based on the consumer price index [19]. Medication costs were based on the Dutch Pharmacotherapeutic Compass [24]. Costs of illness during additional life years were not determined, [13] since the follow-up period was too short to capture potential long-term differences in economic costs related to PTB.

### Statistical analysis

For the main trial, a sample size calculation was performed based on the primary clinical outcome (severe adverse neonatal outcome). An independent sample size calculation for the primary economic outcome (cost effectiveness) was not performed. Analyses were performed by intention-to-treat which was prespecified in the study protocol. Differences in resource use were assessed using the non-parametric Whitney U-test or chi-square test. Costs were calculated by multiplying resource use by unit costs. For twin infants, maternal costs were split equally between them, as this reflects actual clinical practice and resource use (e.g., admission costs per day are similar for a mother with a twin pregnancy as for a mother with a singleton pregnancy) (S1 Table in S1 File supporting information for details). Costs were reported for the total trial period and by phase (antepartum/intrapartum, postpartum before and after discharge).

Missing post-hospital discharge data were expected due to self-reported questionnaires and handled using multiple imputation techniques. The pattern of missingness was explored using logistic regression analysis with ‘missingness’ of costs as the (binary) response variable with both baseline and outcome covariates as explanatory variables and seemed to be at random (i.e., ‘missingness’ independent of the missing and observed outcomes) (S3 Table in S1 File supporting information). Therefore, we used Multiple Imputation by Chained Equations (MICE) with predictive mean matching, stratified for treatment allocation and generated 50 complete datasets [25]. Total costs for the postpartum period after discharge were imputed, after which the total costs (also including the antepartum and postpartum admission period) were passively imputed. Rubin’s rules were used to pool results from 50 different multiply imputed datasets to estimate mean costs with standard errors (SEs) for the atosiban and placebo groups. For each bootstrap replicate, resampled participants (including all 50 of their imputed values) were pooled across imputations using Rubin’s rules, such that the resulting confidence intervals reflect both imputation and sampling uncertainty. To estimate the 95% confidence interval (CI) around the difference in mean costs and effects, non-parametric bootstrapping was used with 5000 replications, based on the normal approximation to the bootstrap distribution [26]. We accounted for the interdependence between twins by treating the mother as the clustering unit, which was implemented using a cluster bootstrap. As randomisation was stratified by site, we planned to incorporate centre into the regression model. However, due to the low number of events in several participating centres, this was not possible. The incremental cost-effectiveness ratio (ICER) was calculated by dividing the difference in mean costs by the difference in mean effects between treatment groups, expressed as incremental cost per severe adverse neonatal outcome prevented. Uncertainty surrounding ICERs was estimated using bootstrapping and visualised by plotting the bootstrapped samples on a cost-effectiveness plane. These statistical analyses were pre-specified as detailed in the APOSTEL 8 trial protocol [13]. Cost-effectiveness acceptability curves were estimated showing the probability that atosiban is cost-effective in comparison with placebo for a range of different willingness to pay (WTP) thresholds for preventing a severe adverse neonatal outcome. As no accepted WTP threshold exists for this outcome and this was not prespecified in the trial protocol, we report the WTP threshold at which the probability of cost-effectiveness of atosiban reached 50% [27].All analyses were performed in Stata/SE 17.0 and SPSS version 20.0 (Chicago, IL, USA).

For sensitivity analysis, a complete-case analysis was performed based on cases for which all data was complete, which was not pre-specified in the trial protocol. Furthermore, subgroup analyses were performed in accordance with the statistical analysis plan for the APOSTEL 8 trial, as no subgroup analyses were specifically for the economic evaluation: composition of pregnancy (singleton and twin pregnancy), ruptured membranes at randomisation (in total, singleton and twin population), cervical length at randomisation (< 15 mm and 15–30 mm), and history of spontaneous PTB (yes and no). A post-hoc sensitivity analysis was performed to assess departures from the missing-at-random assumption, using a pattern-mixture approach on the imputed post-discharge cost values. Given that after-discharge costs did not differ significantly between complete cases and the imputed population within either treatment group, we based the sensitivity parameter on the observed standard deviation of post-discharge costs (approximately €430.0–560.0). Imputed values in both treatment groups were shifted by €0, €250.0, €500.0, €750.0, and €1,000.0 (approximately 0.5–2 standard deviations).

## Results

In total, 755 participants were randomised in the APOSTEL 8 trial of whom 752 had their primary outcome available and were thus included in the current economic evaluation: 375 (449 infants) in the atosiban group and 377 (435) infants in the placebo group. Clinical outcomes did not significantly differ between groups, with a composite adverse neonatal outcome in 37/449 (8.2%) infants in the atosiban group versus 40/435 (9.2%) infants in the placebo group (relative risk (RR) 0.90; 95% CI 0.58 to 1.40, p 0.63). Full details of the clinical outcomes of APOSTEL 8 trial are reported elsewhere [10]. Cost data were complete for all infants up until hospital discharge. Post-discharge cost data were available for 488 infants (atosiban N = 262/449; placebo N = 226/435), since not all participants returned the follow-up questionnaire at three months of corrected child age (Fig 1).

**Fig 1:**
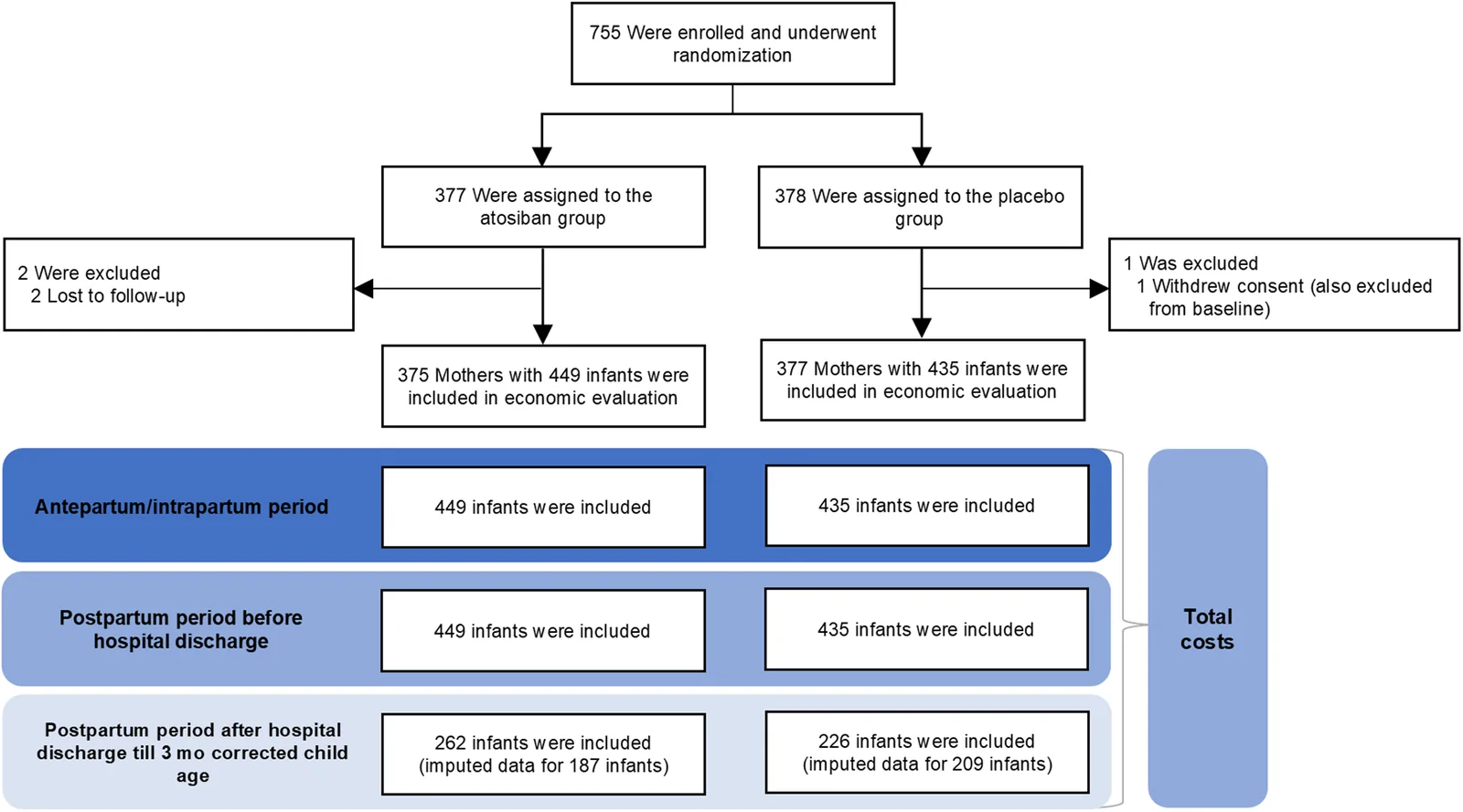
Flow-chart.

### Resource use

Resource use was generally similar between both groups (S2 Table in S1 File supporting information). More vaginal twin births occurred in the atosiban group, reflecting the higher proportion of twin pregnancies compared to the placebo group (17.9% versus 12.0%, p 0.014). The mean duration of hospital admissions did not differ between the groups. After hospital discharge, the atosiban group used less maternity care than the placebo group (mean hours 11.0 versus 15.5, p 0.0020). Other resource use categories did not significantly differ between the trial groups.

### Costs

Total mean antepartum/intrapartum costs were significantly higher in the atosiban group than in the placebo group, with a mean difference (MD) of €500.3 (bootstrap 95% CI €94.1 to €906.3) (Table 1). This was mainly driven by the atosiban costs of €496.6 on average. Postpartum costs during and after hospital did not significantly differ (Tables 1 and 2). After imputing post-discharge costs for the 396 infants without follow-up data, total mean costs including all three periods were generated for all participants. The total mean imputed costs were €40743.1 in the atosiban group and €40793.6 in the placebo group, resulting in a non-significant cost saving of atosiban of €50.5 (bootstrap 95% CI −4037.6 to 3936.7) (Table 3). In the pattern-mixture sensitivity analysis on the imputed post-discharge costs, we found that the estimated cost difference between atosiban and placebo remained cost saving across the full range tested, ranging from −€50.5 (no shift) to −€114.5 (largest shift tested) (S4 Table in S1 File supporting information)

**Table 1:** Costs during hospital admission (ante-/intrapartum and postpartum period).

	Atosiban N = 449	Placebo N = 435		
	Mean (SE) costs (€)		Mean difference (95% CI)	*p-value*
*Antepartum and intrapartum admission period*				
Tocolysis	496.6 (10.6)	0	496.6 (476.0 to 517.3)	<0.001
Medication^a^	14.0 (0.9)	13.8 (0.7)	0.2 (−1.9 to 2.3)	0.85
Delivery^b^	1496.5 (39.6)	1585.4 (44.5)	−88.6 (−206.0 to 28.9)	0.14
Manual placental removal	73.7 (11.4)	55.9 (10.3)	17.77 (−11.8 to 47.4)	0.24
Packed cells	13.8 (4.2)	17.4 (5.2)	−3.67 (−16.7 to 9.4)	0.58
Maternal antepartum admission^c^	2652.5 (127.5)	2574.6 (133.5)	77.87 (−288.1 to 443.8)	0.68
**Total ante-/intrapartum costs**	**4747.4 (141.2)**	**4247.2 (148.4)**	**500.25 (94.2 to 906.3)**	**0.020**
*Postpartum admission period before discharge from hospital*				
Maternal postpartum admission^d^	813.3 (63.8)	761.5 (69.3)	51.9 (−128.4 to 232.2)	0.57
Neonatal admission^e^	26,453.1 (1360.2)	26,118.2 (1419.5)	334.85 (−3498.9 to 4168.6)	0.86
Neonatal operation	15.1 (7.6)	11.7 (6.8)	3.4 (−15.9 to 22.7)	0.73
Neonatal transfer to other hospital	678.4 (58.7)	671.2 (53.7)	7.2 (−147.5 to 161.9)	0.93
**Total postpartum admission costs**	**27,960.0 (1395.4)**	**27,562.7 (1458.3)**	**397.4 (−3534.9 to 4329.6)**	**0.84**
*Antepartum, intrapartum and postpartum admission period*				
**Total antepartum and post-partum admission costs**	**32,707.4 (1382.2)**	**31,809.8 (1431.6)**	**897.6 (−2980.0 to 4775.2)**	**0.65**

All 95% confidence intervals (CI) are calculated using non-parametric bootstrapping. SE, standard error. ^a^Includes corticosteroids, antibiotics and magnesium sulfate for neuroprotection. ^b^ Also includes epidural costs if applicable. ^c^ Includes admission to ward, medium care and home monitoring. ^d^ Includes admission to ward and medium care. ^e^ Includes admission to ward, medium care and intensive care.

**Table 2:** Costs after discharge from hospital based on self-reported questionnaire^a^.

*Costs of complete cases*				
	Atosiban N = 262	Placebo N = 226		
	Mean (SE) costs (€)		Mean difference (95% CI)	*p-value*
Productivity loss^b^	4168.1 (285.4)	4766.1 (320.3)	−598.0 (−1431.7 to 235.7)	0.16
Informal care^c^	1680.0 (240.8)	1762.6 (303.3)	−82.7 (−823.1 to 657.7)	0.83
Homecare^d^	54.36 (35.7)	28.5 (15.8)	25.9 (−50.8 to 102.6)	0.51
Primary care^e^	1021.5 (61.5)	1280.9 (77.6)	−259.4 (−452.3 to −66.5)	0.0080
Outpatient hospital visits^f^	942.6 (87.9)	1021.6 (90.2)	−79.1 (−325.0 to 166.9)	0.53
Diagnostic tests^g^	170.7 (20.6)	207.4 (23.3)	−36.7 (−97.1 to 23.7)	0.23
**Total postpartum after discharge costs**	**8037.1 (467.8)**	**9067.0 (559.3)**	**−1029.9 (−2432.9 to 373.1)**	**0.92**
*Costs after imputation of missing cases*				
	**Atosiban N = 449**	**Placebo N = 435**		
	Mean (SE) costs (€)		Mean difference (95% CI)	*p-value*
**Imputed total postpartum after discharge costs**	**8035.7 (434.8)**	**8983.8 (480.0)**	**−948.1 (−1761.0 to 135.1)**	

All 95% confidence intervals (CI) are calculated using non-parametric bootstrapping. SE, standard error. ^a^ Based on the self-reported iMTA questionnaire sent at three months of corrected child’s age. ^b^ Includes both paid, unpaid and educational maternal work loss costs starting from trial entry. ^c^ Includes unpaid care provided by family members, friends or other individuals. ^d^ Includes paid homecare for cleaning, selfcare support and nursing. Travel costs are included. ^e^ Includes maternity care, and consultations of both mother and child with a general practitioner, midwife, physiotherapist, social worker, dietician, homeopathic physician and speech therapist. Travel costs are included. ^f^ Includes consultations to gynecologist, pediatrician, child revalidation specialist, psychologist or psychiatrist, other medical specialists (e.g., cardiologist) and emergency room visits of both mother and child. Travel costs are included. ^g^ Includes diagnostic tests performed in both mother and child; CT-scan, MRI, ultrasounds, brainscan and renogram.

**Table 3:** Total costs and cost-effectiveness.

	Total costs (€), mean (SE)		Incremental costs (95% CI)	Absence of severe adverse neonatal outcome, mean (SE)		Incremental health gain (95% CI)	ICER
	Atosiban	Placebo		Atosiban	Placebo		
*Base-case analysis*							
	**N = 449**	**N = 435**		**N = 449**	**N = 435**		
**Total group (imputed)**	**40,743.1 (1458.7)**	**40,793.6 (1494.5)**	**−50.5 (−4037.6 to 3936.7)**	**0.92 (0.013)**	**0.91 (0.014)**	**0.0093 (−0.029 to 0.048)**	**Dominant (−5286.6**)
*Subgroup analysis*							
	N = 301	N = 319		N = 301	N = 319		
Singleton pregnancies	40,396.8 (1866.6)	40,007.2 (1748.3)	389.6 (−4485.0 to 5264.2)	0.92 (0.016)	0.89 (0.017)	0.027 (−0.018 to 0.072)	14,510.1
	N = 148	N = 116		N = 148	N = 116		
Twin pregnancies	41,447.5 (2284.7)	42,956.2 (2887.1)	−1508.7 (−8423.2 to 5405.8)	0.91 (0.023)	0.95 (0.21)	−0.036 (−0.10 to 0.02)	41,776.7
	N = 125	N = 124		N = 125	N = 124		
PPROM at inclusion	55,403.9 (2718.5)	54,095.7 (2887.0)	1308.2 (−6466.2 to 9082.6)	0.84 (0.032)	0.86 (0.031)	−0.019 (−0.11 to 0.07)	Dominated (−68,302.9)
	N = 321	N = 311		N = 321	N = 311		
NO PPROM at inclusion	34,897.1 (1621.1)	35,489.9 (1657.9)	−592.8 (−4915.5 to 3729.9)	0.95 (0.013)	0.93 (0.015)	0.021 (−0.017 to 0.059)	Dominant (−28,234.6)
*Sensitivity analysis*							
	N = 262	N = 226		N = 262	N = 226		
Complete-case analysis	45,851.3 (2076.7)	40,017.25 (1957.7)	5834.1 (185.8 to 11482.4)	0.91 (0.018)	0.90 (0.020)	0.0019 (−0.051 to 0.055)	3030.2

All 95% confidence intervals (CI) are calculated using non-parametric bootstrapping. SE, standard deviation. ICER, incremental cost-effectiveness ratio. PPROM, preterm prelabor rupture of membranes. Severe adverse neonatal outcome: perinatal mortality, bronchopulmonary dysplasia, periventricular leukomalacia > grade I, intraventricular haemorrhage > grade II, necrotising enterocolitis > stage I, retinopathy of prematurity > grade II or laser therapy and/or culture proven sepsis.

### Cost-effectiveness

The cost-effectiveness analysis used the proportion of infants without a severe adverse neonatal outcome as the health outcome. Atosiban showed a non-significant health gain over placebo (0.92 versus 0.91; incremental gain 0.0093, bootstrap 95% CI –0.060 to 0.078). Dividing the mean cost difference by the mean effect difference yielded an ICER of -€5286.6. This point estimate falls in the south-east quadrant of the cost-effectiveness plane, indicating that the intervention was on average dominant (both cost-saving and more effective, on average), although this classification was based on point estimates alone (Table 3). As illustrated by the cost-effectiveness plane, there was substantial uncertainty surrounding the incremental cost and effect estimates (Fig 2). Each dot represents the incremental costs and effects difference for a bootstrapped sample of treatment with atosiban compared to placebo. The bootstrapped cost-effect pairs were distributed across all four quadrants of the cost-effectiveness plane: 41% in the south-east quadrant (less costly and more effective), 29% in the north-east quadrant (more costly and more effective), 20% in the north-west quadrant (more costly and less effective), and 11% in the south-west quadrant (less costly and less effective). This distribution across all four quadrants indicates substantial uncertainty regarding the cost-effectiveness of atosiban compared with placebo. The probability of cost-effectiveness reached 50% at a WTP threshold of €33,023.3.. Probabilities of cost-effectiveness across a broad range of thresholds can be observed in the cost-effectiveness acceptability curve (Fig 3). Based on the complete-case analysis, the ICER was €3030.2 (north-east quadrant) and the probability of cost-effectiveness reached 50% at a WTP threshold of €3,032,84.0 (Table 3, S3 Fig in S1 File supporting information).

**Fig 2:**
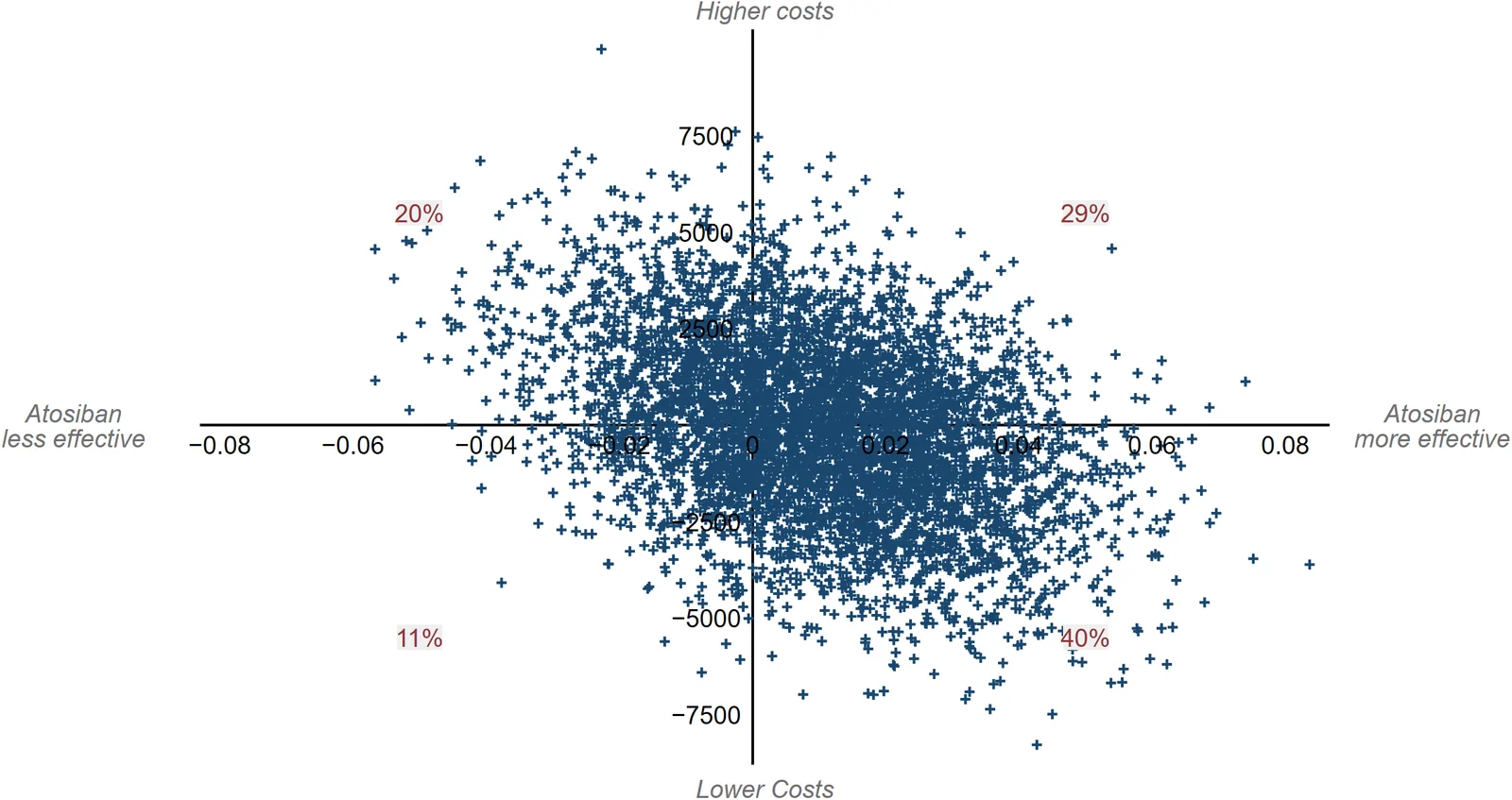
Cost-effectiveness plane – total group. Each point in the cost-effectiveness plane represents incremental costs and health gain of treatment with atosiban compared with placebo (5000 samples from multiply imputed datasets).

**Fig 3:**
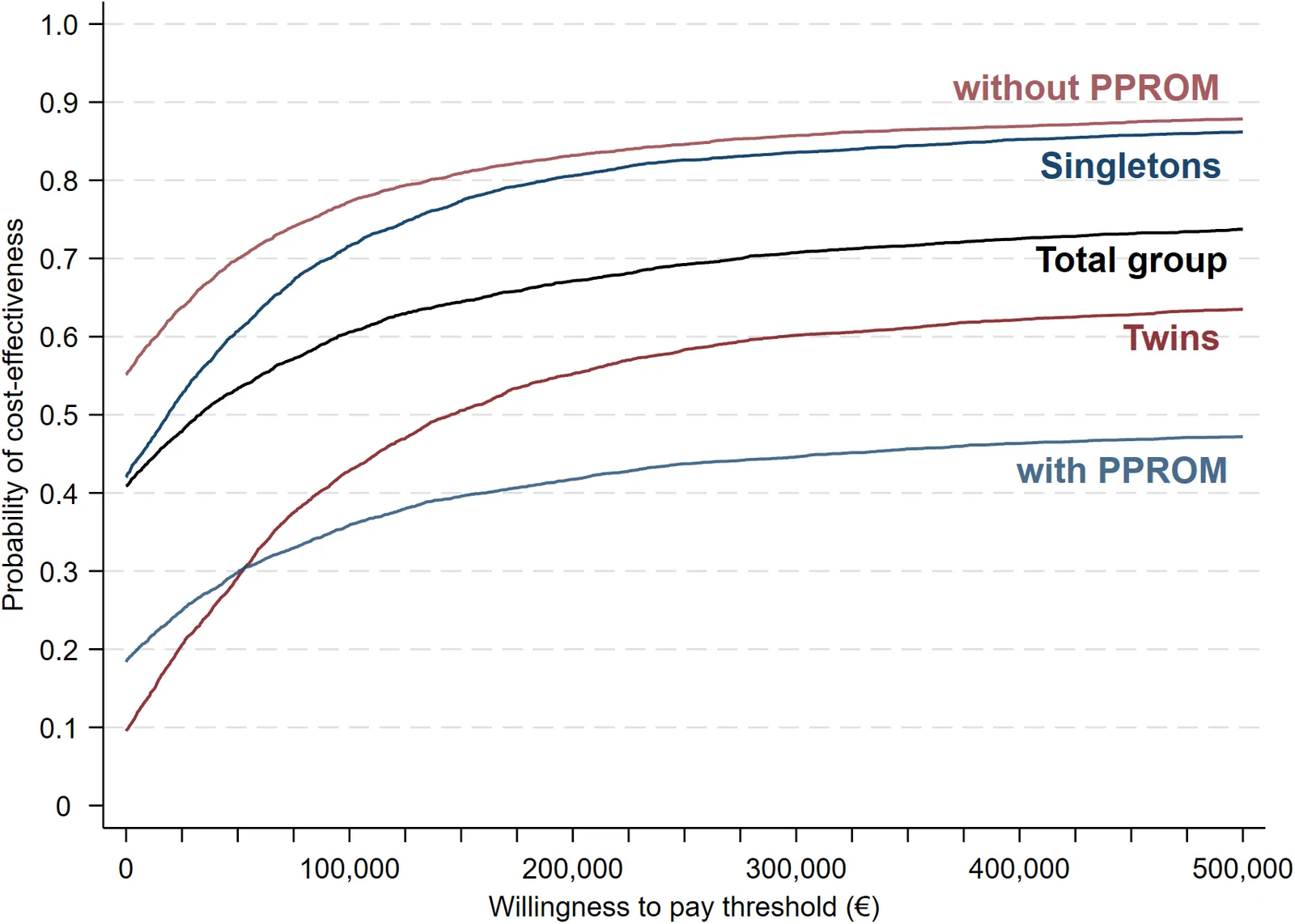
Cost-acceptability curves for total and subgroups. The probability of atosiban being cost effective compared with placebo at different WTP thresholds.

### Subgroup analyses

In singleton pregnancies, 88% of bootstrapped cost-effectiveness pairs were spread across the north-east and south-east quadrants in the cost-effectiveness plane, indicating that atosiban was more effective in preventing severe adverse neonatal outcomes than placebo in the majority of replications, irrespective of whether this was accompanied by lower or higher costs (Fig 4, blue). The cost-effectiveness acceptability curve shows that the probability of cost-effectiveness reached 50% at a WTP of €18,809.0 (Fig 3). In twin pregnancies, this was €145,020.2 (Fig 4, red). In pregnancies with ruptured membranes, both the cost-effectiveness plane and cost-effectiveness acceptability curve demonstrated that atosiban was not cost-effective compared to placebo (Figs 3 and 5, blue). However, in pregnancies with unruptured membranes, 55% of the bootstrapped cost-effectiveness pairs were located in the south-east quadrant of the cost-effectiveness plane, indicating that atosiban was, on average, more often cost saving and more effective than placebo (Fig 5, red). The probability of cost-effectiveness therefore exceeded 50% at a WTP of €0 (Fig 3). In singleton pregnancies with unruptured membranes, 96% of bootstrapped estimates were spread across the north-east and south-east quadrants of the cost-effectiveness plane and the probability of cost-effectiveness reached 50% at a WTP of €11,445.9 (S5 Table and S1 Fig in S1 File supporting information)

**Fig 4:**
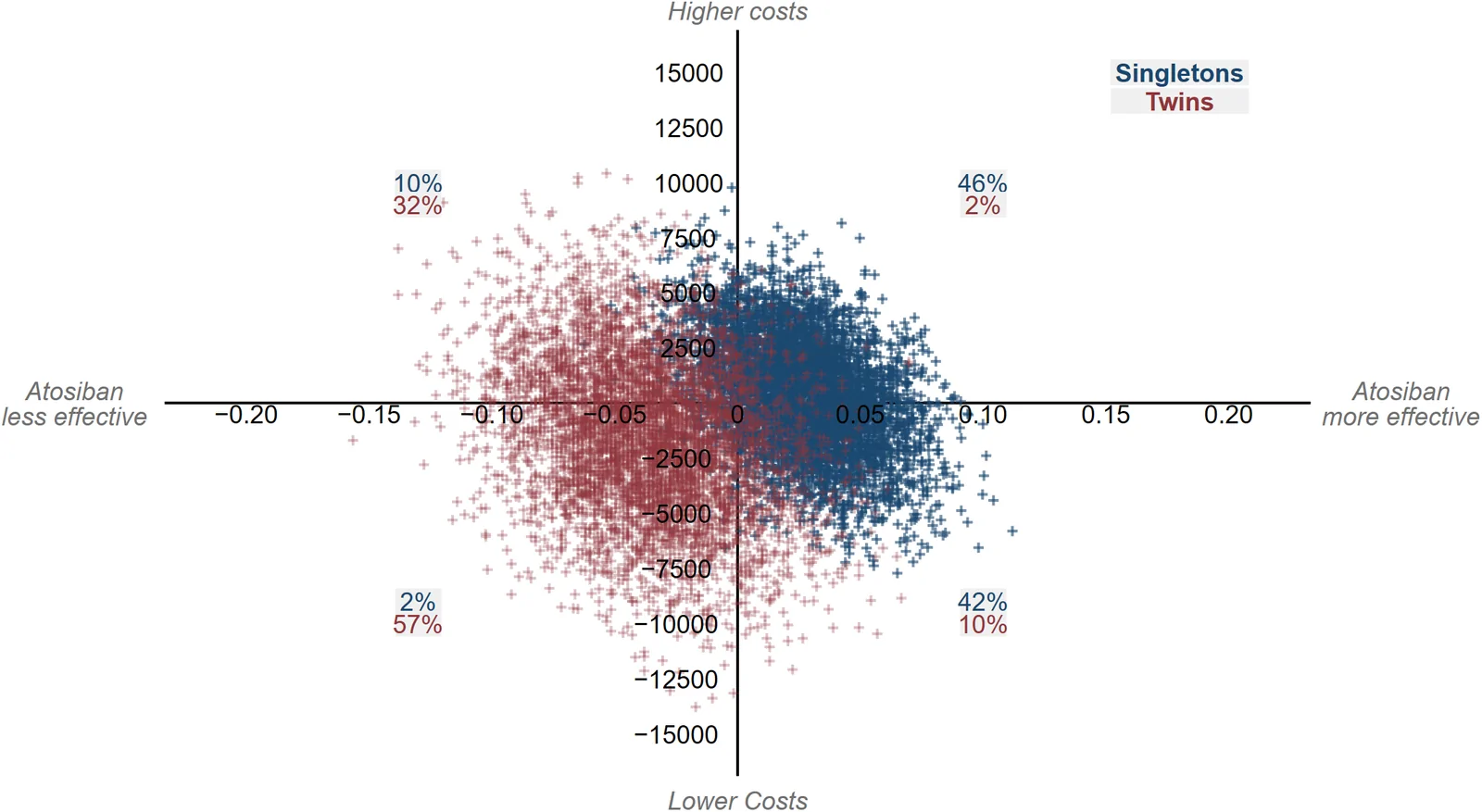
Cost-effectiveness plane – subgroups singleton and twin pregnancies. Subgroup singletons is blue. Subgroup twins is red. Each point in the cost-effectiveness plane represents incremental costs and health gain of treatment with atosiban compared with placebo (5000 samples from multiply imputed datasets).

**Fig 5:**
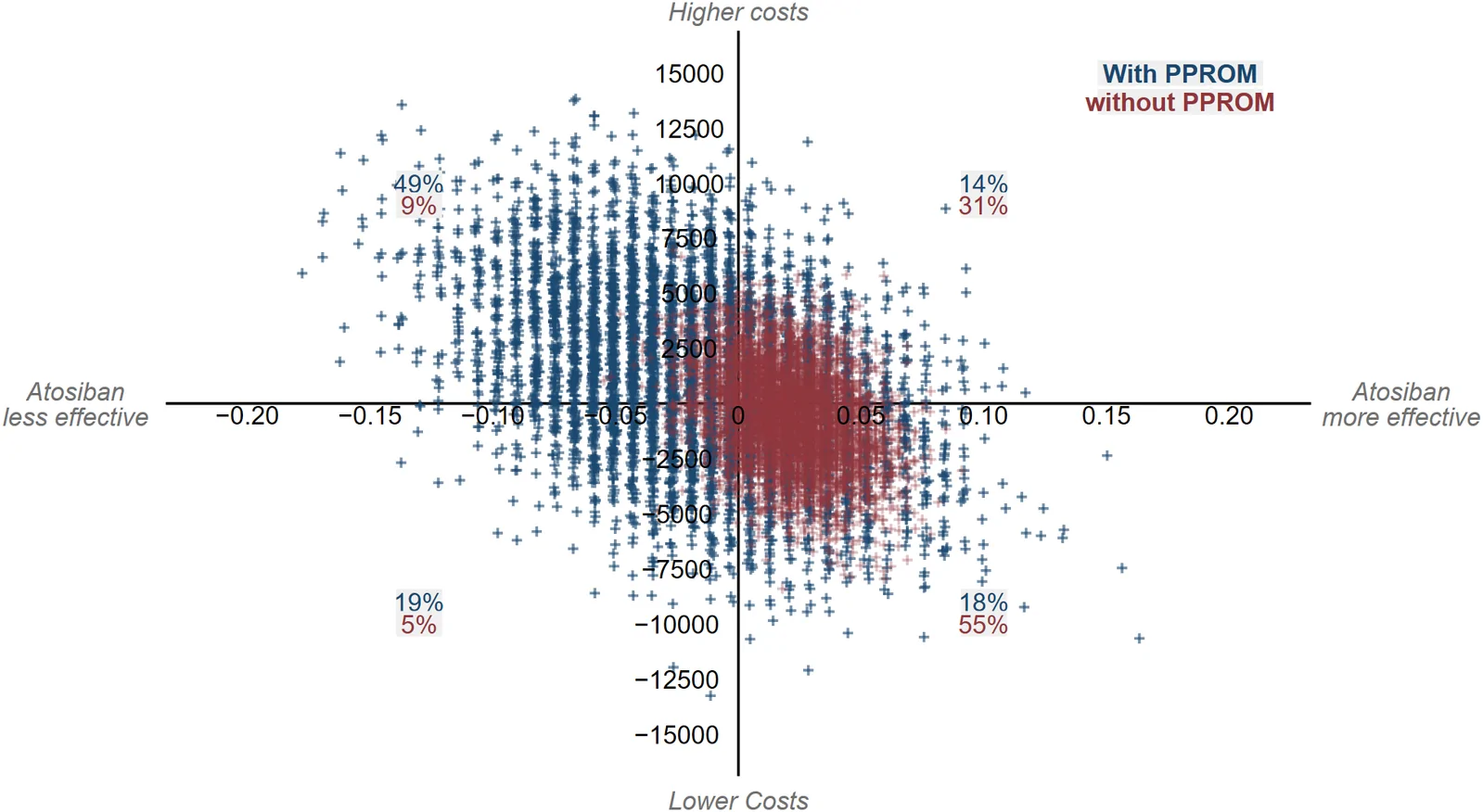
Cost-effectiveness plane – subgroup with and without ruptured membranes at inclusion. PPROM denotes preterm prelabor rupture of membranes. Subgroup with PPROM is blue. Subgroup without PPROM is red. Each point in the cost-effectiveness plane represents incremental costs and health gain of treatment with atosiban compared with placebo (5000 samples from multiply imputed datasets).

## Discussion

### Main findings

In this alongside economic evaluation of the double-blind randomised controlled APOSTEL 8 trial, we assessed the cost-effectiveness of atosiban compared to placebo as tocolytic treatment for threatened PTB from 30^+0^ to 33^+6^ weeks of gestation. The health outcome was absence of a severe adverse neonatal outcome, which did not significantly differ between the trial groups. Total mean costs, estimated from a societal perspective and collected up until three months of corrected child age, were comparable between the trial groups, with a non-significant mean cost saving of €50 for atosiban. The ratio of differences in mean costs and mean effects resulted in a negative ICER, corresponding to the south-east (dominant) quadrant of the cost-effectiveness plane and indicating that, on average, atosiban is both cost saving and more effective. However, in the cost-effectiveness plane, bootstrapped cost-effect pairs were distributed over all four quadrants, indicating substantial uncertainty surrounding the cost-effectiveness of atosiban. Furthermore, the probability of cost-effectiveness of atosiban reached 50% at a WTP of €33,023.3. Therefore, atosiban is unlikely to be a cost-effective treatment for threatened PTB from 30^+0^ to 33^+6^ weeks’ gestation. In singleton pregnancies, atosiban may be cost-effective compared to placebo, specifically in those with unruptured membranes. In twin pregnancies and in pregnancies with ruptured membranes, we observed that cost-effectiveness of atosiban compared to placebo was absent.

### Strengths and limitations

The current study was based on a pragmatic randomised trial including a large sample size and diverse study population from both academic and secondary care hospitals [13]. Therefore our findings are likely generalisable to populations of healthcare systems organised similarly to the Netherlands. Due to the nature of the study, data on resource use was collected prospectively, ensuring comprehensive and accurate cost tracking across a wide range of resource categories. The analysis was conducted from a societal perspective, as recommended by health economic evaluation guidelines [19]. Furthermore, we used a validated economic questionnaire to generate key economic estimates, enhancing the reliability of our data. Our methods can provide guidance on future health economic modelling. A recent meta-analysis on cost-effectiveness of antenatal corticosteroids and tocolytics pointed out that further robust cost-effectiveness studies are needed for these critical interventions [28]. To our knowledge, this is the largest and most extensive economic evaluation on tocolytic treatment to date, thereby significantly contributing to the total evidence base.

However, there were also limitations. Since we adopted a societal perspective, cost data for the postpartum period after hospital discharge relied on participant questionnaires. This resulted in high levels of missing data during this phase (43% in the atosiban group and 49% in the placebo group). The complete-case analysis showed a substantially lower probability of cost-effectiveness of atosiban than the primary imputed analysis. This discrepancy may partly reflect bias in the complete-case estimates: among completers in the atosiban group, mean postpartum costs post-discharge were €33,199.5, while this was €27,960.0 among all participants allocated to atosiban. In addition, the complete-case analysis excluded nearly half of participants, which may have disrupted the balance across treatment arms achieved through randomisation. We used multiple imputation to address this, leveraging the complete data to inform imputed postpartum costs [29]. However, there remains residual uncertainty as to whether the assumptions underlying the imputation method are fully met, which should be considered when interpreting our estimates. Also, the time horizon of our study was short with a follow-up period up to three months corrected child age. Therefore, lifelong societal and educational costs that may be associated with severe neonatal morbidities are not captured.

Another limitation concerns the use of severe adverse neonatal outcomes prevented as the effect measure, which was based on the primary outcome for the underlying clinical trial. For this outcome, no reference cost values exist. Nevertheless, as shown by the cost-effectiveness acceptability curve, even at a very high WTP threshold of €500,000 to prevent a severe adverse neonatal outcome, the probability of cost-effectiveness of atosiban did not reach 80%. Moreover, a related limitation is that comparisons of cost-effectiveness with other health conditions are problematic due to the disease specific nature of the outcome. To account for this, the quality-adjusted life year (QALY) is routinely used as a health outcome measure within health economic evaluations [30]. However, in our case this would have required the application of a validated preference-based outcome measure for infants which is currently non-existent [31]. Finally, our follow-up period until three months of corrected age of the infant is likely to be too short to capture long-term differences in health outcomes related to PTB. Health economic modelling can be used to extrapolate the outcomes of the current study to long-term outcomes.

### Interpretation

To our knowledge this is the first economic evaluation of atosiban compared to placebo as a treatment for threatened PTB, challenging the comparison of our findings with previous literature. In the economic evaluation of the randomised APOSTEL 3 study, cost-effectiveness of nifedipine and atosiban were compared for threatened PTB between 25–34 weeks of gestation [27]. Although costs in the APOSTEL 3 study were collected over a shorter time period (the first six weeks postpartum), total mean costs of atosiban were almost €3,000 higher than in our study. This difference was mainly driven by the higher neonatal admission costs in the APOSTEL 3 study, likely due to the lower gestational age at inclusion of the trial participants, resulting in more infants born extremely preterm with longer neonatal admissions.

In our study, the cost-effectiveness outcomes were remarkedly different after stratifying for singleton and twin pregnancies. This was mainly due to a higher rate of severe adverse neonatal outcome in twin pregnancies in the atosiban group compared to the placebo group. Although a direct comparison of our cost-effectiveness findings with those of the APOSTEL 3 study is not possible as it compared atosiban with nifedipine, the findings for singleton and twin pregnancies also differed notably in the APOSTEL 3 study. This suggests that both the costs and effects of tocolytics are different in singleton and twin pregnancies, potentially necessitating separate evaluations in future trials.

Examining cost-effectiveness more closely in singleton pregnancies, in those with unruptured membranes, the probability of cost-effectiveness reached 95% at the highest examined hypothetical WTP threshold of €500,000, suggesting a potential economic benefit of atosiban in this subgroup. However, no interaction test or multiplicity correction was applied to this estimate. Formal interaction testing for the underlying clinical outcome showed no significant treatment-by-subgroup effect (P-interaction = 0.14–0.26), suggesting this finding should be considered hypothesis-generating. We therefore recommend that future adequately powered studies further assess the (cost-)effectiveness of atosiban in women with singleton pregnancies with unruptured membranes. Also, a key question remains: what is the WTP threshold to prevent one severe adverse neonatal outcome? This has yet to be established by stated preference. A well-defined WTP threshold specific to management of PTB will strengthen future economic evaluations and guide clinical decision-making. Lastly, must be noted that this economic evaluation was not independently powered for precision of cost-effectiveness estimates. Given the small, imprecise observed effect difference and wide cost confidence interval, our findings should be interpreted primarily through the uncertainty intervals and cost-effectiveness acceptability curves presented.

## Conclusions

This economic evaluation did not demonstrate that atosiban is cost-effective compared to placebo for the treatment of threatened PTB from 30^+0^ to 33^+6^ weeks gestation. Apart from the lack of clinical benefit, our findings show no economic advantage, further discouraging its routine use. These results contribute to optimizing evidence-based management of threatened preterm birth.

## Supporting information

S1 File S1 Table. Resources and unit costs. S2 Table. Resource use per treatment group. S3 Table. Imputation model. S4 Table. Pattern-mixture sensitivity analysis. S5 Table. Total costs and cost-effectiveness – subgroup analysis. S6 Table. Costs per trial period – complete-case analysis. S1 Fig. Cost-effectiveness plane (top) and cost-acceptability curve (bottom) – subgroups singleton with preterm prelabor rupture of membranes (PPROM) and without PPRROM. S2 Fig. Cost-effectiveness plane (top) and cost-acceptability curve (bottom) – subgroups twins with preterm prelabor rupture of membranes (PPROM) and without PPRROM. S3 Fig. Cost-effectiveness plane (top) and cost-acceptability curve (bottom) – complete case analysis.
(ZIP)
