# Supplementary material for: Cost-effectiveness of atosiban versus placebo for threatened preterm birth (APOSTEL 8 trial)
Source: PLoS One. 2026 Nov 5;21(11):e0347475. doi: 10.1371/journal.pone.0347475 (PMC13739115; doi:10.1371/journal.pone.0347475)
Supplement: Supplementary file 1 [file pone.0347475.s001.zip › Supporting information/S1 Table. Resources and unit costs.pdf]

Table S1. Resources and unit costs

Type of costs	Unit	Unit cost	Valuation method (source)	Volume source
**Medical costs**				
Atosiban bolus*	treatment	€ 26,26	Dutch Pharmacotherapeutic Compass	CRF
Atosiban high dose infusion (18mg/h, max 3h)*	per hour	€ 38,89	Dutch Pharmacotherapeutic Compass	CRF
Atosiban low dose infusion (6 mg/h,max 45h)*	per hour	€ 12,96	Dutch Pharmacotherapeutic Compass	CRF
Atosiban complete course*	treatment	€ 700,10	Dutch Pharmacotherapeutic Compass	CRF
Corticosteroids*	per gift	€ 5,58	Dutch Pharmacotherapeutic Compass	CRF
Magnesium sulfate bolus*	treatment	€ 17,25	Dutch Pharmacotherapeutic Compass	CRF
Magnesium sulfate continued (1gr/h)*	per hour	€ 3,45	Dutch Pharmacotherapeutic Compass	CRF
Antibiotics prophylactic during labor (every 6h)[a]*	per gift	€ 2,29	Dutch Pharmacotherapeutic Compass	CRF
Antibiotics suspected infection during labor starting dose[b]*	treatment	€ 33,00	Dutch Pharmacotherapeutic Compass	CRF
Antibiotics peripartum suspected infection continued (every 6h)[c]*	per gift	€ 3,25	Dutch Pharmacotherapeutic Compass	CRF
Antibiotics peripartum endocarditis[d]*	treatment	€ 6,50	Dutch Pharmacotherapeutic Compass	CRF
Epidural*	procedure	€ 93,63	Top-down calculation	CRF
Vaginal delivery spontaneous singleton	procedure	€ 1.006,23	Top-down calculation	CRF
Vaginal delivery spontaneous twin*	procedure	€ 2.164,79	Top-down calculation	CRF
Vaginal delivery breech	procedure	€ 1.517,59	Top-down calculation	CRF
Vaginal delivery instrumental	procedure	€ 1.471,45	Top-down calculation	CRF
Caesarean section*	procedure	€ 3.591,35	Top-down calculation	CRF
Manual placental removal*	procedure	€ 973,43	Top-down calculation	CRF
Erythrocyte transfusion*	per gift	€ 280,83	Top-down calculation	CRF
Antepartum home monitoring*	per day	€ 319,64	Top-down calculation	CRF
Maternal admission ward*	per day	€ 766,89	Top-down calculation	CRF
Maternal admission medium care*	per day	€ 817,15	Top-down calculation	CRF
Neonatal admission ward	per day	€ 996,63	Top-down calculation	CRF
Neonatal admission medium care	per day	€ 996,63	Top-down calculation	CRF
Neonatal admission intensive care	per day	€ 3.056,57	Top-down calculation	CRF
Neonatal transfer to other hospital	per drive	€ 1.578,33	Top-down calculation	CRF
Noenatal surgical procedure	procedure	€ 1.700,00	Top-down calculation	CRF
Homecare cleaning*	per hour	€ 34,00	Dutch costing guideline	iMTA
Homecare personal*	per hour	€ 59,77	Dutch costing guideline	iMTA
Homecare nursing*	per hour	€ 77,85	Dutch costing guideline	iMTA
Homecare traveling*	per visit	€ 31,49	Dutch costing guideline	iMTA
Maternal care*	per hour	€ 59,33	Dutch costing guideline	iMTA
Midwife postpartum care*	treatment	€ 395,35	Dutch costing guideline	iMTA
General practitioner[d]	per visit	€ 36,38	Dutch costing guideline	iMTA
Physiotherapist[d]*	per visit	€ 45,03	Dutch costing guideline	iMTA
Social worker*	per visit	€ 131,83	Dutch costing guideline	iMTA
Dietician*	per visit	€ 25,64	Dutch costing guideline	iMTA
Preverbal speech therapist	per visit	€ 42,49	Dutch costing guideline	iMTA

Homeopath/osteopath	per visit	€ 108,67	Private practice	iMTA
Psychologist/psychotherapist/prychiatrist*	per visit	€ 125,60	Dutch costing guideline	iMTA
Gynaecologist*	per visit	€ 207,98	Top-down calculation	iMTA
Other medical specialist	per visit	€ 217,06	Top-down calculation	iMTA
Paediatrician	per visit	€ 276,93	Top-down calculation	iMTA
Child revalidation specialist	per visit	€ 515,89	Dutch costing guideline	iMTA
Emergency room mother*	per visit	€ 400,00	Top-down calculation	iMTA
Emergency room child	per visit	€ 400,00	Top-down calculation	iMTA
Ambulance to hospital from home[d]	per visit	€ 681,97	Dutch costing guideline	iMTA
Maternal ultrasound*	per procedure	€ 123,81	Top-down calculation	iMTA
Maternal MRI*	per procedure	€ 277,15	Dutch costing guideline	iMTA
Maternal CT-scan*	per procedure	€ 212,79	Dutch costing guideline	iMTA
Neonatal brain MRI	per procedure	€ 283,79	Top-down calculation	iMTA
Neonatal X-ray	per procedure	€ 62,99	Top-down calculation	CRF
Neonatal brain ultrasound	per procedure	€ 161,52	Top-down calculation	CRF
Neonatal renogram	per procedure	€ 169,18	Top-down calculation	iMTA
**Non-medical costs**				
Tavel to hospital including parking costs	per visit	€ 5,20	Dutch costing guideline	iMTA
Travel to general practitioner including parking costs	per visit	€ 4,34	Dutch costing guideline	iMTA
Travel to physiotherapist including parking costs*	per visit	€ 4,66	Dutch costing guideline	iMTA
Informal care*	per hour	€ 19,51	Dutch costing guideline	iMTA
Work loss (absentism)*	per hour	€ 40,36	Dutch costing guideline	iMTA
Unpayed work loss*	per hour	€ 19,51	Dutch costing guideline	iMTA
Education loss*	per hour	€ 24,00	Dutch costing guideline	iMTA

CRF, Case Record Form. iMTA Productivity Cost Questionnaire sent at three months corrected child's age.
All unit costs are from 2023. In the top-down calculation costs are an average of an academic and general hospital.
* Costs are split between children of a twin pregnancy
[a] Penicillin intravenously
[b] Gentamicin and amoxicillin intravenously
[c] Amoxicillin intravenously
[d] Visits of both mother and child
