# Supplementary material for: Cost-effectiveness of atosiban versus placebo for threatened preterm birth (APOSTEL 8 trial)
Source: PLoS One. 2026 Nov 5;21(11):e0347475. doi: 10.1371/journal.pone.0347475 (PMC13739115; doi:10.1371/journal.pone.0347475)
Supplement: Supplementary file 1 [file pone.0347475.s001.zip › Supporting information/S2 Table. Resource use per treatment group.pdf]

Table S2. Resource use per treatment group

	Atosiban (N=449)				Placebo (N=435)				
	% patients using care	Mean volume[a]	Mean volume[b]	Mean (SE) costs (€)	% patients using care	Mean volume[a]	Mean volume[b]	Mean (SE) costs (€)	p-value[b]
Antenatal period									
Medication									
Atosiban bolus (treatment)	99.1		NA		NA	0	0	0	<0.001
Atosiban high dose infusion (hours)	99.1	2.46 (1.12)	2.44 (1.14)		NA	0	0	0	<0.001
Atosiban low dose infusion (hours)	94.1	31.98 (18.77)	30.19 (19.66)	496.64 (224.73)[c]	NA	0	0	0	<0.001
Corticosteroids (gifts)	98.2	1.42 (0.76)	1.43 (0.78)	7.95 (3.26)	98.4	1.43 (072)	1.41 (0.73)	7.88 (3.31)	0.79
Magnesium sulfate bolus (treatment)	4.0		NA		5.1				0.45
Magnesium sulfate (hours)	4.0	8.41 (14.22)	0.34 (3.23)	1.74 (13.04)[d]	5.1	4.80 (6.46)	0.24 (1.77)	1.63 (8.52)[b]	0.59
Antibiotics prophylactic during labor (gifts)	20.3	3.43 (2.73)	0.69 (1.88)	1.59 (4.30)	27.6	2.70 (1.66)	0.74 (1.56)	1.71 (3.57)	0.66
Antibiotics suspected infection during labor (gifts)	8.0	3.27 (3.85)	0.13 (0.99)	2.63 (10.23)	6.7	5.32 (4.16)	0.17 (1.18)	2.53 (10.52)	0.67
Antibiotics for endocarditis prophylaxis (treatment)	0.9		NA	0.015 (0.31)	0.5		NA	0.043 (0.53)	0.44
Epidural (procedure)	20.5		NA	19.18 (34.87)	20.0		NA	18.73 (34.70)	0.87
Delivery									
Vaginal spontaneous singleton (procedure)	48.6		NA	488.55 (503.47)	51.5		NA	518.15 (503.47)	0.38
Vaginal spontaneous twin (procedure)	17.8		NA	192.86 (414.65)	12.0		NA	129.39 (351.56)	0.014
Vaginal breech (procedure)	2.4		NA	37.18 (234.87)	3.0		NA	45.35 (258.70)	0.62
Vaginal instrumental (procedure)	6.5		NA	95.04 (362.08)	5.5		NA	81.06 (335.84)	0.56

Cesarean (procedure)	24.7		NA	664.01 (1243.00)	28.0		NA	792.69 (1355.45)	0.26
Manual placenta removal (procedure)	9.4		NA	73.71 (240.85)	6.9		NA	55.94 (214.45)	0.18
Erythrocyt transfusion (gifts)	2.4	2.00 (0.45)	0.05 (0.32)	0.00 (0.00)	2.8	2.25 (0.75)	0.6 (0.39)	0.00 (0.00)	NA
Maternal admission									
Ward (days)	82.9	3.64 (3.73)	3.02 (3.66)	2314.33 (2806.96)	88.0	3.27 (3.76)	3.01 (4.29)	2209.00 (2822.73)	0.58
Medium Care (days)	14.3	2.64 (2.51)	0.38 (1.32)	307.57 (1077.80)	12.6	3.25 (3.7)	0.41 (1.69)	336.25 (380.87)	0.73
Home monitoring (days)	0.9	10.75 (13.50)	0.10 (1.50)	30.61 (478.72)	1.1	8.00 (11.79)	0.09 (1.42)	29.39 (453.16)	0.97
Postpartum period during admission before discharge									
Maternal admission									
Ward (days)	36.3	2.90 (2.50)	1.05 (2.05)	807.88 (1572.96)	34.5	2.78 (2.57)	0.96 (2.00)	735.16 (1536.10)	0.49
Medium Care (days)	0.7	1.00 (1.00)	0.01 (0.11)	5.46 (86.15)	0.9	3.50 (2.9)	0.03 (0.41)	26.30 (336.39)	0.20
Neonatal admission									
Ward (days)	40.3	16.82 (15.09)	6.78 (12.64)	6756.66 (12595.15)	36.1	16.98 (15.24)	6.13 (12.25)	6108.08 (12212.81)	0.44
Medium Care (days)	44.7	22.80 (15.31)	10.21 (15.28)	10172.73 (15227.15)	46.2	23.19 (14.77)	10.71 (15.31)	10678.83 (15261.16)	0.62
Intensive Care (days)	33.4	9.33 (9.29)	3.12 (6.93)	9523.70 (21195.23)	33.8	9.03 (10.21)	3.05 (7.30)	9331.32 (22326.43)	0.90
Neonatal transfer to other hospital (drives)	32.1	1.34 (0.85)	0.43 (0.79)	678.44 (1243.40)	33.6	1.27 (0.66)	0.43 (0.71)	671.24 (1119.44)	0.93
Neonatal surgical procedure (procedure)	0.9			15.14 (159.92)	0.7			11.72 (140.85)	0.74
Postpartum period period after discharge based on self-reported questionnaire (only of participant who filled in the questionnaire)									
	**Atosiban N=262**				**Placebo N=226**				
	% patients using care	Mean volume[a]	Mean volume[b]	Mean (SD) costs (€)	% patients using care	Mean volume[a]	Mean volume[b]	Mean (SD) costs (€)	
Paid workloss (hours)	46.3	100.38 (74.71)	88.74 (78.78)	3183.82 (3136.42)	41.4	111.42 (72.53)	78.89 (77.71)	3581.47 (3179.54)	0.41

Unpaid workloss (hours)	10.0	246.98 (305.89)	42.42 (156.47)	827.61 (3052.82)	10.1	266.18 (334.20)	51.82 (180.29)	1011.07 (3517.38)	0.54
Education workloss (hours)	2.2	190.00 (199.92)	6.53 (49.27)	156.64 (1182.40)	2.1	163.40 (178.51)	7.23 (49.08)	173.52 (1177.91)	0.88
Informal care (hours)	16.5	304.86 (273.96)	86.11 (199.75)	1679.95 (3897.16)	16.1	291.69 (344.27)	90.35 (233.70)	1762.63 (4559.54)	0.83
Homecare cleaning (hours)	0.7	49.67 (43.59)	0.57 (6.53)	19.34 (221.89)	0.7	36.00 (31.18)	0.48 (5.07)	16.25 (172.33)	0.87
Homecare selfcare (hours)	0.9	22.25 (35.55)	0.34 (4.69)	20.30 (280.32)	0.9	12.00 (0.00)	0.11 (1.13)	6.35 (67.32)	0.47
Homecare nursing (hours)	0.7	6.67 (0.58)	0.08 (0.71)	5.94 (55.46)	0.0	0.00 (0.00)	0.00 (0.00)	0.00 (0.00)	0.11
Maternity care (hours)	33.6	19.00 (15.06)	10.95 (14.79)	649.57 (877.55)	31.7	25.39 (17.17)	15.50 (18.26)	919.69 (1083.37)	0.0020
Midwife postpartum care (treatment)	50.4		NA	162.97 (175.58)	46.0		NA	153.94 (178.24)	0.57
Visits general practitioner	24.5	3.61 (3.23)	1.52 (2.74)	55.13 (99.83)	20.7	3.53 (2.67)	1.41 (2.42)	51.19 (87.87)	0.65
Visits physiotherapist	20.0	4.28 (4.29)	1.47 (3.23)	66.17 (145.32)	18.9	3.83 (3.01)	1.39 (2.58)	62.56 (116.20)	0.77
Visits social worker	8.7	3.03 (2.61)	0.45 (1.47)	59.37 (193.42)	8.7	3.11 (2.06)	0.52 (1.43)	68.83 (188.78)	0.59
Visits dietician	1.1	2.20 (1.30)	0.04 (0.34)	1.08 (8.77)	1.1	3.60 (3.90)	.08 (0.74)	2.04 (19.04)	0.46
Visits homeopath /osteopath	5.1	2.09 (1.22)	0.18 (0.69)	19.91 (74.97)	5.3	1.83 (1.14)	0.19 (0.66)	20.20 (71.61)	0.97
Visits preverbal speech therapist	3.3	3.00 (2.85)	0.17 (0.96)	7.30 (40.85)	1.8	1.63 (0.99)	0.06 (0.35)	2.44 (14.79)	
Visits psychologist/ psychotherapist/psychiatrist	8.2	2.78 (3.22)	0.39 (1.54)	49.39 (193.66)	6.4	4.32 (3.35)	0.54 (1.84)	67.25 (231.10)	0.35
Outpatient visits gynecologist	14.9	2.55 (4.32)	0.65 (2.44)	135.74 (508.29)	15.4	3.19 (3.08)	0.95 (2.22)	196.94 (461.71)	0.17
Outpatient visits other medical specialist by mother	4.5	1.75 (2.14)	0.13 (0.74)	29.00 (160.85)	5.1	3.14 (3.14)	0.31 (1.34)	66.27 (290.47)	0.08
Outpatient visits pediatrician	34.1	3.01 (4.14)	1.76 (3.49)	487.27 (966.77)	27.6	3.25 (3.57)	1.73 (3.06)	477.89 (847.94)	0.91
Outpatient visits child revalidation specialist	0.2	1.00 (NA)	0.004 (0.06)	1.97 (31.87)	0.2	1.00 (NA)	.004 (0.07)	2.28 (34.32)	0.92
Maternal ultrasound (procedure)	8.0	2.89 (2.23)	0.40 (1.29)	49.15 (159.60)	12.0	3.69 (2.80)	0.85 (2.05)	105.18 (253.99)	0.0030
Maternal MRI (procedure)	0.2	1.00 (NA)	0.004 (0.06)	1.06 (17.12)	0.0	0.00 (0.00)	0.00 (0.00)	0.00 (0.00)	0.35
Maternal CT-scan (procedure)	0.0	0.00 (0.00)	0.00 (0.00)	0.00 (0.00)	0.2	1.00 (NA)	0.004 (0.07)	0.94 (14.15)	0.28

Neonatal MRI (procedure)	0.4	1.50 (0.71)	0.01 (0.14)	3.24 (39.01)	0.7	1.00 (00)	0.009 (0.08)	2.50 (22.95)	0.80
Neonatal X-ray (procedure)	0.9	1.00 (00)	0.015 (0.12)	0.96 (7.72)	0.2	1.00 (NA)	0.004 (0.067)	0.28 (4.18)	0.24
Neonatal brain ultrasound (procedure)	17.8	2.36 (2.42)	0.72 (1.72)	116.52 (277.82)	16.1	2.03 (2.08)	0.61 (1.47)	98.44 (236.99)	0.44
Neonatal renogram (procedure)	0.0	0.00 (0.00)	0.00 (0.00)	0.00 (0.00)	0.5	1.50 (0.71)	0.013 (0.15)	2.25 (1.67)	0.07
Visits emergency room	6.2	1.25 (0.67)	0.13 (0.44)	53.44 (177.38)	9.9	1.16 (0.70)	0.22 (0.55)	88.45 (219.80)	0.06
Ambulance (number)	9.1	1.61 (1.43)	0.25 (0.81)	171.79 (552.61)	6.0	1.35 (0.70)	0.15 (0.49)	105.61 (334.34)	0.12
Travel times to hospital	42.3	3.71 (4.93)	2.69 (4.51)	13.97 (23.46)	36.8	4.59 (4.95)	3.25 (4.65)	16.89 (24.20)	0.18

[a] Only including participants using care.

[b] Including all participants.

[c] Includes all atosiban costs (so atosiban bolus, high dose infusion and low dose infusion).

[d] Includes all magnesium sulfate costs (so magnesium sulfate bolus and infusion)
