# Supplementary material for: Cost-effectiveness of atosiban versus placebo for threatened preterm birth (APOSTEL 8 trial)
Source: PLoS One. 2026 Nov 5;21(11):e0347475. doi: 10.1371/journal.pone.0347475 (PMC13739115; doi:10.1371/journal.pone.0347475)
Supplement: Supplementary file 1 [file pone.0347475.s001.zip › Supporting information/S3 Figure. Cost-effectiveness plane (top) and cost-acceptability curve (bottom) – complete case analysis.pdf]

Figure S3. Cost-effectiveness plane (top) and cost-acceptability curve (bottom) – complete case analysis

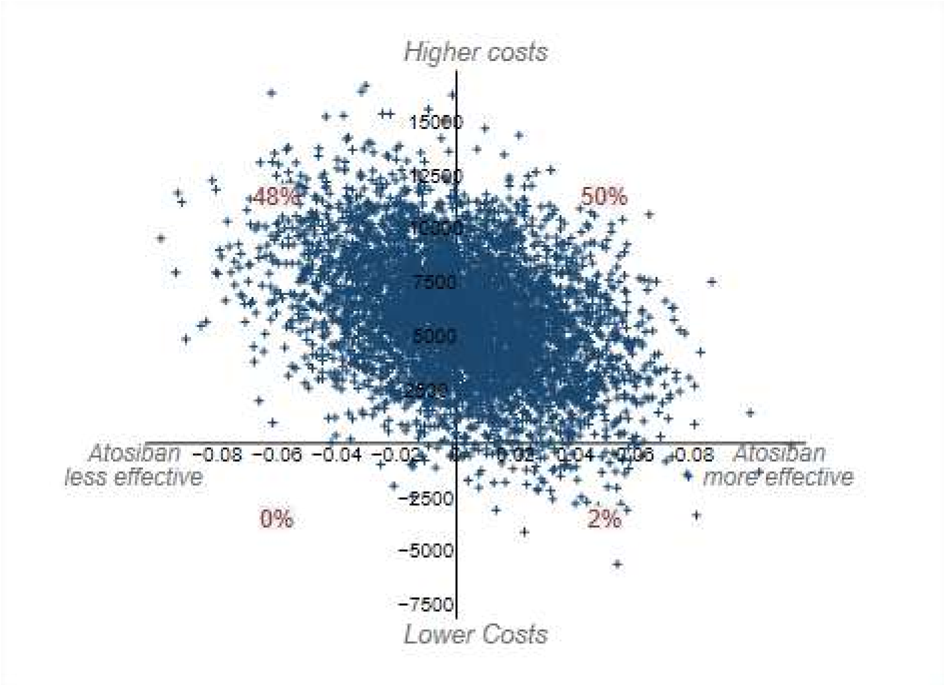

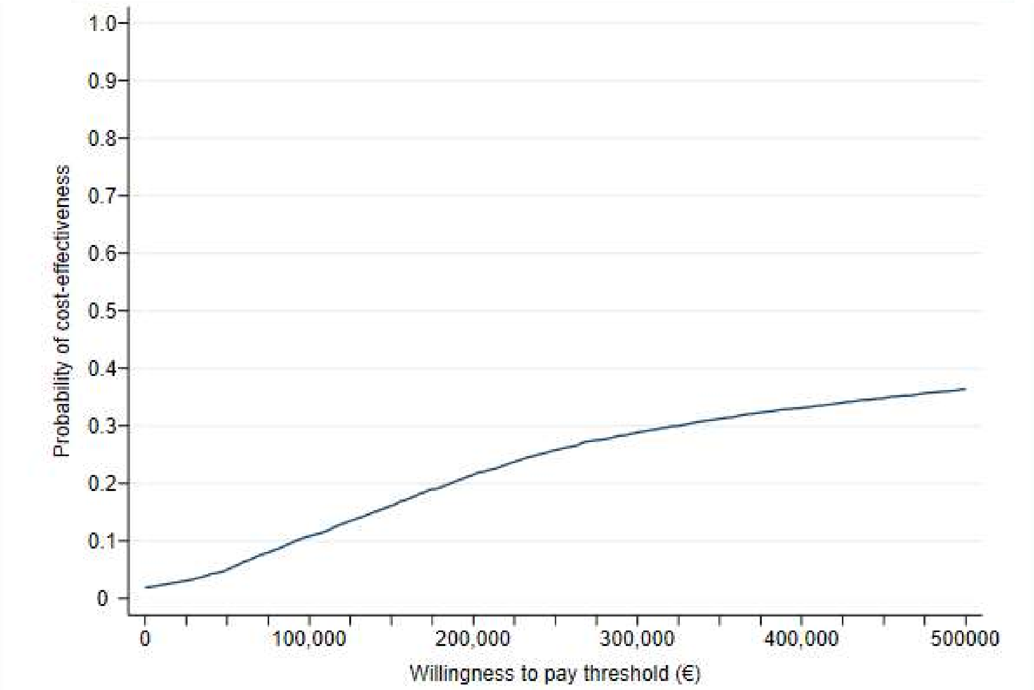

Each point in the cost-effectiveness plane (top) represents incremental costs and health gain of treatment with atosiban compared with placebo (5000 samples).

The cost-acceptability curve (bottom) shows the probability of atosiban meing cost effective compared with placebo at different WTP thresholds.
