# Supplementary material for: Cost-effectiveness of atosiban versus placebo for threatened preterm birth (APOSTEL 8 trial)
Source: PLoS One. 2026 Nov 5;21(11):e0347475. doi: 10.1371/journal.pone.0347475 (PMC13739115; doi:10.1371/journal.pone.0347475)
Supplement: Supplementary file 1 [file pone.0347475.s001.zip › Supporting information/S3 Table. Imputation model.pdf]

Table S3. Imputation model

Variable	Role in imputation model
Total costs after discharge	Imputed (outcome variable)
Maternal ethnicity (white vs. other)	Imputed (predictor with missing data)
Baseline working hours per week	Imputed (predictor with missing data)
Participant ID	Regular (identifier)
Maternal age	Regular (predictor)
Singleton/twin status (composition of pregnancy)	Regular (predictor)
Gestational age at randomization	Regular (predictor)
Gestational age at birth	Regular (predictor)
Neonatal sex	Regular (predictor)
Neonatal composite outcome (severe adverse outcome)	Regular (predictor)
Total costs until discharge from hospital (so including the antepartum, intrapartum, postpartum admission costs which were CRF-derived)	Regular (predictor)

Ruptured membrane status was not included as a separate predictor, as it correlates strongly with the neonatal composite outcome (already included in the model), given that neonatal sepsis is a component of the composite outcome.
