# Supplementary material for: Cost-effectiveness of atosiban versus placebo for threatened preterm birth (APOSTEL 8 trial)
Source: PLoS One. 2026 Nov 5;21(11):e0347475. doi: 10.1371/journal.pone.0347475 (PMC13739115; doi:10.1371/journal.pone.0347475)
Supplement: Supplementary file 1 [file pone.0347475.s001.zip › Supporting information/S4 Table. Pattern-mixture sensitivity analysis.pdf]

Table S4. Pattern-mixture sensitivity analysis

Delta applied (€)	Total mean cost placebo (€)	Total mean cost atosiban (€)	Difference in costs (€)
250.0	40913.7	40847.2	-66.5
500.0	41033.8	40951.4	-82.5
750.0	41153.9	41055.5	-98.5
1000.0	41274.1	41159.6	-114.5
