# Supplementary material for: Cost-effectiveness of atosiban versus placebo for threatened preterm birth (APOSTEL 8 trial)
Source: PLoS One. 2026 Nov 5;21(11):e0347475. doi: 10.1371/journal.pone.0347475 (PMC13739115; doi:10.1371/journal.pone.0347475)
Supplement: Supplementary file 1 [file pone.0347475.s001.zip › Supporting information/S5 Table. Total costs and cost-effectiveness – subgroup analysis.pdf]

Table S5. Total costs and cost-effectiveness per subgroup

	Total costs (€), mean (SE)		Incremental costs (95% CI)	Absence of severe adverse neonatal outcome, mean (SE)		Incremental health gain (95% CI)	ICER
	**Atosiban**	**Placebo**		**Atosiban**	**Placebo**		
Singleton with PPROM at inclusion*	N=84<br>52,791.2 (3797.8)	N=88<br>52,800.1 (3652.0)	-8.9 (-9963.3 to 9945.6)	N=84<br>0.83 (0.041)	N=88<br>0.84 (0.039)	-0.0076 (-0.12 to 0.10)	1170.9
Singleton without PPROM at inclusion*	N=217<br>35,598.9 (2045.0)	N=231<br>35,133.7 (1886.9)	465.2 (-4764.2 to 5694.6)	N=217<br>0.95 (0.014)	N=231<br>0.91 (0.018)	0.040 (-0.0054 to 0.086)	11,487.6
Twin with PPROM*	N=44<br>60,391.7 (3065.0)	N=36<br>57,262.6 (4400.7)	3129.1 (-7050.6 to 13308.8)	N=44<br>0.86 (0.052)	N=36<br>0.92 (0.047)	-0.053 (-0.19 to 0.080)	Dominated (-59,005.5)
Twin without PPROM*	N=84<br>33,432.7 (2616.2)	N=80<br>36,518.4 (3447.3)	-3085.7 (-11156.2 to 4984.8)	N=84<br>0.93 (0.025)	N=80<br>0.96 (0.021)	-0.030 (-0.091 to .031)	103,519.7
Cervical length <15 mm at inclusion	N=166<br>41,200.1 (2511.8)	N=173<br>41,835.5 (2452.8)	-635.3 (-7249.4 to 5978.7)	N=166<br>0.92 (0.021)	N=173<br>0.90 (0.023)	0.026 (-0.036 to 0.087)	Dominant (-24,689.7)
Cervical length 15–30 mm at inclusion	N=155<br>28,146.7 (1842.0)	N=138<br>27,534.9 (1918.5)	611.8 (-4310.5 to 5534.2)	N=155<br>0.97 (0.013)	N=138<br>0.96 (0.016)	0.0104 (-0.028 to 0.49)	58,685.1
Previous PTB	N=55<br>39,466.7 (5447.3)	N=59<br>39,678.9 (3940.4)	-212.3	N=55<br>0.89 (0.042)	N=59<br>0.92 (0.037)	-0.024	8718.9
No previous PTB	N=394<br>40,921.3 (1481.6)	N=376<br>40,968.5 (1615.8)	-47.2 (-4252.8 to 4158.4)	0.92 (0.014)	0.91 (0.015)	0.014 (-0.025 to 0.054)	Dominant (-3276.9)

All 95% confidence intervals (CI) are calculated using non-parametric bootstrapping. SE, standard error. PPROM, preterm prelabor rupture of membranes.
Severe adverse neonatal outcome: perinatal mortality, bronchopulmonary dysplasia, periventricular leukomalacia > grade I, intraventricular haemorrhage > grade II, necrotising enterocolitis > stage I, retinopathy of prematurity > grade II or laser therapy and/or culture proven sepsis.

* Included in figure S1 and S2
