# Supplementary material for: Cost-effectiveness of atosiban versus placebo for threatened preterm birth (APOSTEL 8 trial)
Source: PLoS One. 2026 Nov 5;21(11):e0347475. doi: 10.1371/journal.pone.0347475 (PMC13739115; doi:10.1371/journal.pone.0347475)
Supplement: Supplementary file 1 [file pone.0347475.s001.zip › Supporting information/S6 Table. Costs per trial period – complete-case analysis.pdf]

Table S6. Costs during all study periods – complete case analysis

	Atosiban N=262	Placebo N=226		
	Mean (SE) costs (€)		Mean difference (95% CI)	*p-value*
Antepartum and intrapartum period				
Tocolysis	489.5 (13.8)	0	489.5 (462.2 to 516.8)	<0.001
Medication[a]	14.4 (1.3)	13.8 (0.9)	0.6 (-2.5 to 3.8)	0.70
Delivery[b]	1496.5 (1.9)	1602.6 (63.6)	-106.2 (-268.8 to 56.4)	0.19
Manual placental removal	70.6 (14.5)	68.9 (15.8)	1.7 (-40.3 to 43.6)	0.94
Packed cells	15.0 (5.8)	18.6 (7.9)	-3.6 (-22.8 to 15.5)	0.71
Maternal antepartum admission[c]	2528.8 (140.6)	2739.3 (171.5)	-210.6 (-647.2 to 225.9)	0.34
**Total antepartum costs**	**4614.7 (162.3)**	**4443.4 (193.9)**	**171.4 (-327.6 to 670.3)**	**0.50**
Postpartum period during admission before discharge from hospital				
Maternal postpartum admission[d]	793.4 (78.3)	788.8 (108.6)	4.6 (-255.8 to 265.1)	0.97
Neonatal admission[e]	31554.8 (1964.7)	25179.8 (1900.3)	6375.0 (1058.9 to 11691.2)	0.02
Neonatal operation	26.0 (12.9)	7.5 (7.5)	-18.4 (-10.9 to 47.8)	0.24
Neonatal transfer to other hospital	825.3 (85.7)	530.8 (61.2)	294.5 (89.9 to 499.2)	0.0070
**Total postpartum during admission costs**	**33199.5 (2012.1)**	**26506.9 (1935.3)**	**6692.6 (1258.8 to 12126.4)**	**0.02**
Postpartum period after discharge from hospital based on self-reported questionnaire				
**Total postpartum after discharge costs**	**8037.1 (467.8)**	**9067.0 (559.3)**	**-1029.9 (-2432.9 to 373.1)**	**0.92**
Total period				
**Total costs**	**45851.3 (2076.7)**	**40017.3 (1957.7)**	**5834.1 (185.8 to 11482.4)**	**0.040**

All 95% confidence intervals (CI) are calculated using non-parametric bootstrapping . SE, standard error.

[a] Includes corticosteroids, antibiotics and magnesium sulfate for neuroprotection.

[b] Also includes epidural costs if applicable.

[c] Includes admission to ward, medium care and home monitoring.

[d] Includes admission to ward and medium care.

[e] Includes admission to ward, medium care and intensive care.
